# Histone Deacetylase Inhibitors Enhance Cell Killing and Block Interferon-Beta Synthesis Elicited by Infection with an Oncolytic Parainfluenza Virus

**DOI:** 10.3390/v11050431

**Published:** 2019-05-10

**Authors:** Candace R. Fox, Griffith D. Parks

**Affiliations:** Burnett School of Biomedical Sciences, College of Medicine, University of Central Florida, Orlando, FL 32827, USA; candace.fox@ucf.edu

**Keywords:** parainfluenza virus, oncolytic virus, histone deacetylase inhibitors

## Abstract

Previous results have shown that infection with the cytoplasmic-replicating parainfluenza virus 5 mutant P/V-CPI- sensitizes cells to DNA damaging agents, resulting in the enhanced killing of airway cancer cells. Here, we have tested the hypothesis that histone deacetylase (HDAC) inhibitors can also act with P/V-CPI- infection to enhance cancer cell killing. Using human small cell lung cancer and laryngeal cancer cell lines, 10 HDAC inhibitors were tested for their effect on viability of P/V-CPI- infected cells. HDAC inhibitors such as scriptaid enhanced caspase-3/7, -8 and -9 activity induced by P/V-CPI- and overall cell toxicity. Scriptaid-mediated enhanced killing was eliminated in lung cancer cells that were engineered to express a protein which sequesters double stranded RNA. Scriptaid also enhanced cancer cell killing by two other negative strand RNA viruses – the La Crosse virus and vesicular stomatitis virus. Scriptaid treatment enhanced the spread of the P/V-CPI- virus through a population of cancer cells, and suppressed interferon-beta induction through blocking phosphorylation and nuclear translocation of Interferon Regulatory Factor 3 (IRF-3). Taken together, these data support a role for combinations of a cytoplasmic-replicating RNA virus such as the P/V-CPI- mutant along with chemotherapeutic agents.

## 1. Introduction

Oncolytic viruses have shown to be promising treatments for human cancers. The recent FDA approval of a modified herpes simplex virus type 1 for the treatment of advanced malignant melanoma [1], and the number of recombinant viruses in development are evidence of strong interest in these therapies for a variety of cancers [2,3]. A range of paramyxoviruses have been proposed as oncolytic vectors due to their inherent cytopathic properties and their ability to activate the immune response, including measles virus, Newcastle disease virus, Sendai virus, and mumps virus [4,5,6,7,8,9,10]. Based on our prior work with an oncolytic Parainfluenza virus type 5 (PIV5), the overall goal of the work described here was to test the hypothesis that selective killing of human airway cancer cells can be enhanced by combinations of chemotherapy and oncolytic virus infection.

Wild type (WT) PIV5 is inherently non-cytopathic and is a poor inducer of host cell responses in most human cell types [11,12,13,14], and thus would not be expected to have appropriate oncolytic properties. Non-cytopathic WT PIV5 can be converted into a virus that induces cell killing through engineered substitutions in the viral P/V gene [15,16]. The PIV5 P/V gene encodes the phosphoprotein P and the V protein [17], which share an identical 164 residue amino-terminal domain (the shared P/V region) but have unique C terminal domains. The P protein is an essential subunit of the viral RNA-dependent RNA polymerase [17]. The V protein contains a COOH-terminal cysteine-rich (cys-rich) zinc-binding domain that is required for many V protein functions [18], including blocking type I interferon (IFN) signaling by targeting STAT1 for degradation [19] and inhibiting interferon-beta (*IFN-β*) gene expression through binding to mda-5 [20]. Amino acid substitutions in the PIV5 shared P/V region resulted in a mutant (P/V-CPI-) which overexpresses viral RNA and protein, is a potent inducer of IFN-β and proinflammatory cytokines, is defective in blocking IFN signaling, and induces cell death in cancer cells [14,15,16,21,22,23]. The P/V-CPI- mutant is very effective at killing cancer cells, a property which may be linked to the high induction of double stranded RNA (dsRNA) during virus replication, the shut off of host and viral protein synthesis through Protein Kinase R (PKR) pathways [24], and the activation of caspase dependent death pathways [15,25]. Importantly, previous work has shown that the P/V-CPI- mutant is restricted for tissue culture growth in normal primary prostate cells [21] and can reduce prostate cancer tumor burden in a mouse model system [23].

While the P/V-CPI- virus is inherently cytopathic to cancer cells, we have recently shown that P/V-CPI- infection can sensitize cancer cells to further killing by treatment with cisplatin [26], a DNA adduct-inducing chemotherapy drug. Remarkably, infection with the cytoplasmic-replicating P/V-CPI- virus followed by cisplatin treatment led to increased damage to cellular DNA, along with enhanced caspase activation and death compared to treatment with the virus or drug alone [26]. The ability of P/V-CPI- to sensitize cells to cisplatin-induced death correlated with virus-induced defects in the cell’s ability to repair damaged DNA. These results with DNA-damaging agents, such as cisplatin, raise the question of whether the P/V-CPI- virus would be more effective at killing cancer cells when coupled with other chemotherapeutic agents that alter DNA metabolism and stress responses.

There are 18 known human histone deacetylase (HDAC) enzymes which are involved in a number of cellular processes. HDAC expression levels have been found to be associated with differing prognosis in various cancers. For example, high expression of HDAC1 and HDAC2 has been correlated with a poor prognosis in patients with lung or oral cancers [27,28]. As such, there has been a recent focus on developing HDAC inhibitors for cancer therapy. These chemicals can have pleotropic effects on cancer cells such as altering the cell cycle, inducing senescence or autophagy, altering signaling pathways and immune responses, or regulating apoptosis. Importantly however, many HDAC inhibitors have been shown to have little cytotoxic effect on normal cells [29]. To date, four HDAC inhibitors are approved by the FDA for use in humans (Vorinostat, Romidepsin, Belinostat, and Panobinostat), and a large number of different inhibitors are in clinical trials for cancer therapy [30].

Here we show that treatment of human airway cancer cells with HDAC inhibitors increased cell death and caspase activation induced by P/V-CPI- mutant infection, through pathways that involved at least in part recognition of double stranded RNA. In addition, HDAC inhibitors reduced IFN-β production by P/V-CPI- infection by blocking phosphorylation and nuclear localization of the key transcription factor interferon regulatory factor-3 (IRF-3), resulting in enhanced P/V-CPI- spread through a population of human airway cancer cells. Our studies support a common property by which a cytoplasmic-replicating RNA virus sensitizes cancer cells to chemotherapeutic agents and suggest combined paramyxovirus and chemotherapy as promising future approaches to treat cancer.

## 2. Materials and Methods

### 2.1. Cells, Viruses, and Infections

The H1299 cell line was obtained from Annette Khaled (University of Central Florida, Orlando, FL, USA). A549, HEp-2, Vero, MDBK, and CV-1 cell lines were provided by Robert Lamb (Northwestern University, Evanston, Illinois, USA). Cultures of H1299 non-small cell lung carcinoma cells were grown in Roswell Park Memorial Institute medium (RPMI 1640) supplemented with 10% heat inactivated fetal calf serum (HI FBS, Gibco, Thermo Fisher Scientific, Waltham, MA, USA). Cultures of A549 alveolar adenocarcinoma cells, HEp-2 laryngeal carcinoma cells, Vero cells, MDBK cells, and CV-1 cells were grown in Dulbecco modified Eagle medium (DMEM) supplemented with 10% HI FBS. Previously described A549 cells that constitutively express reovirus type 3 Dearing sigma3 protein [31] were generated by transfection with pCXN-S4T3D [32] followed by selection in DMEM containing 0.5 mg/mL G418. 

Wild type (WT) PIV5 was grown in MDBK cells and titered on CV-1 cells. The P/V mutant rPIV5-P/V-CPI− (P/V-CPI-) encoding Green Flourescence Protein (GFP) as an additional gene between HN and L was generated and grown in Vero cells as described previously [16] using a cDNA plasmid [33] kindly provided by Robert Lamb (Northwestern University, Evanston, Illinois, USA) and Biao He (University of Georgia, Athens, Georgia, USA). P/V-CPI- encodes six naturally-occurring mutations in the amino-terminal region of the P/V gene, resulting in amino acid changes at: Y26H, V32I, T33I, L50P, L102P, and S157F [15]. Vesicular stomatitis virus (VSV), human parainfluenza virus type 2 (hPIV2), and Zika virus (ZIKV, MR766, BEI resources, Manassas, VA, USA) were grown in Vero cells. La Crosse virus (LACV; a kind gift from Andy Pekosz, Johns Hopkins University, Baltimore, Maryland, USA) were grown in C636 cells and stocks were titered by plaque assay on Vero cells.

Infections were performed by incubating virus and cells in DMEM or RPMI supplemented with 10% BSA. After one hour of incubation, cells were washed, and media was replaced with DMEM or RPMI supplemented with 2% HI FBS and various HDAC inhibitors as indicated in the figure legends.

### 2.2. Chemical Preparation

Chemicals were purchased from Sigma-Aldrich (St. Louis, MO, USA). Scriptaid, CI-994, Apicidin, Panobinostat (LBH589), Suberoylanilide hydroxamic acid (SAHA, also known as Vorinostat), Tubacin, and Trichostatin A were reconstituted in sterile dimethyl sulfoxide (DMSO). Sodium 4 Phenylbutuyrate, Suberoyl bis-hydroxamic acid (SBHA), and Valproic Acid were reconstituted in sterile water.

### 2.3. Cell Viability and Caspase Assays

[3–5-dimethylthiazol-2-yl)-5-(3-carboxymethoxy phenyl)-2-(4-sulfophenyl)-2H-tetrazolium salt] MTT cell viability assays were performed in 96-well dishes using Cell Titer 96 Aqueous One solution reagent (Promega, Madison, WI, USA) according to the manufacturer’s instructions. Data are expressed as a percentage of mock-infected cells analyzed in parallel.

Alternatively, cells cultured in 24-well plates (2 cm diameter) were treated as indicated in each figure legend (concentration of drug and time). Media and trypsinized adherent cells were centrifuged and analyzed for annexin V binding (BD Bioscience, San Jose, CA, USA) and propidium iodide (BD Bioscience) staining as described by the manufacturer. Cells were analyzed by flow cytometry using the CytoFLEX (Beckman Coulter, Brea, CA, USA) and 10,000 independent events were analyzed using CytExpert software (Beckman Coulter).

Cytotoxicity assays were performed in 96-well white plates (Corning, Corning, NY, USA) using CytoTox-Glo reagent (Promega) according to the manufacturer’s instructions. Data are expressed as a fold change of mock-infected cells analyzed in parallel. Functional caspase assays were performed in 96-well white plates (Corning) using Caspase-Glo 9, 8, or 3/7 assays (Promega) according to the manufacturer’s instructions. Data are expressed as a fold change of mock-infected cells analyzed in parallel.

### 2.4. Western Blotting

As described in the figure legends, 6-well dishes (60 mm diameter) of cells were treated, followed by lysis in 1X protein lysis buffer (Cell Signaling Technology, Danvers, MA, USA). Cell lysate was resolved on 12% sodium dodecyl sulfate-polyacrylamide gel electrophoresis (SDS-PAGE) gels (Bio-Rad, Hercules, CA, USA) and transferred to nitrocellulose membranes. Samples were probed with antibodies indicated in the figure legends (Cell Signaling Technology), anti-β-actin antibody (A5316, Sigma-Aldrich, St. Louis, MO, USA), or IFIT-1 antibody (Novus Biologicals, Centennial, CO, USA). Blots were visualized by horseradish peroxidase-conjugated antibodies (Cell Signaling Technology) and chemiluminescence (Thermo Fisher Scientific).

### 2.5. Fluorescence Microscopy and IRF-3 immunostaining

Cells were grown on glass bottom 48-well plates (MatTek, Ashland, MA, USA) and treated as indicated in the figure legends. Live imaging microscopy was performed using the Perkin Elmer Ultraview microscope with 20× objective lens. IRF-3 immunostaining was performed as previously described [34] using a primary antibody against IRF-3 at 1:400 dilution (BD PharMingen, clone SL-12.1). Slides were imaged on a Ziess710 confocal microscope with 40× objective lens.

### 2.6. Human IFN-β ELISA

As described in the figure legends, 6 -well dishes (60 mm diameter) of cells were treated and supernatants were evaluated using a VeriKine Human IFN-β ELISA kit as described by the manufacturer (PBL Assay Science, Piscataway, NJ, USA). ELISA results were normalized to 106 cells.

### 2.7. Reverse Transcription and Real Time PCR

As described in the figure legends, 6-well dishes (60 mm diameter) of cells were treated, followed by RNA extraction using TRIzol (Invitrogen, Carlsbad, CA, USA). To produce cDNA, 1 μg of total RNA was used by utilizing TaqMan^®^ Reverse Transcription Reagents (Applied Biosystems, Foster City, CA, USA) as per the manufacturer’s instructions. Quantitative real-time PCR was performed using Bio-Rad CFX Connect Real-Time and Fast SYBR^®^ FAST Green Master Mix (Applied Biosystems). Primers used include: β-actin forward 5′- GATCATTGCTCCTCCTGAGC-3′, and β-actin reverse 5′-ACTCCTGCTTGCTGATCCAC-3′ and OAS2 forward: 5’-AGAAGCTGGGTTGGTTTATC-3’, and OAS2 reverse 5’- GACGTCACAGATGGTGTTC-3’. IFIT1 and TLR3 forward and reverse primers were obtained from studies by [35] and [36], respectively. Relative gene expression was determined using CFX Manager 3.1 Software (Bio-Rad).

### 2.8. Statistical Analyses

Values are the mean of three replicates and experiments were performed at least twice. Statistical analysis was performed using Prism GraphPad, students T test or a two-way ANOVA. In all figures, * indicates *p*-value < 0.05, ** indicates *p*-value < 0.01, and *** indicates *p*-value < 0.001.

## 3. Results

### 3.1. HDAC Inhibitors Enhance Killing of Lung Cancer Cells by the P/V-CPI- Mutant through Increases in Caspase Activity

**Figure 1:**
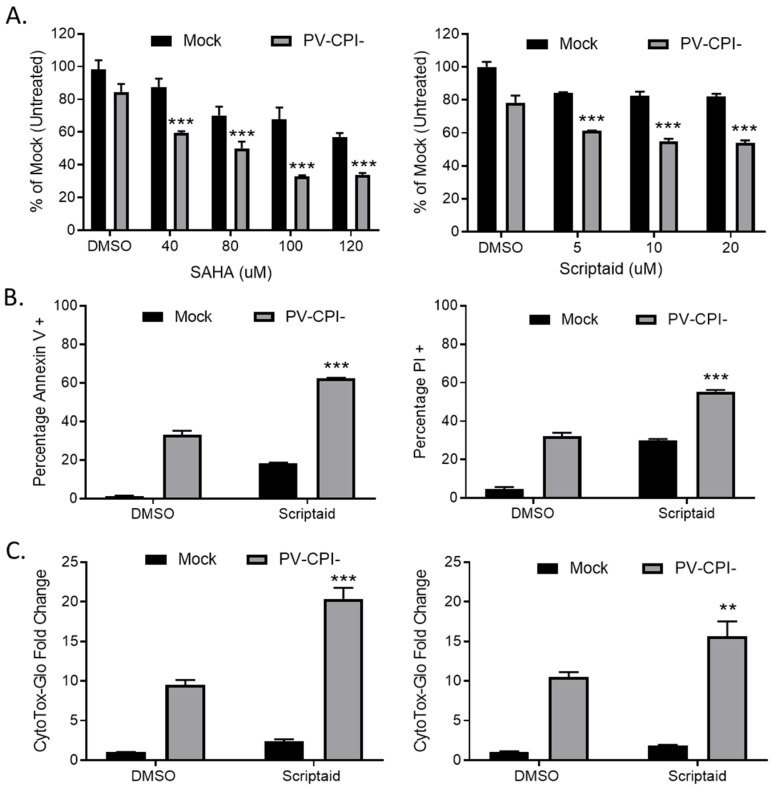
Pretreatment with HDAC inhibitors enhances cell death by the P/V-CPI- mutant virus. (**A**) H1299 cells were pretreated with the indicated concentrations of SAHA (left panel), scriptaid (right panel), or DMSO as a vehicle control for 12 h. Cells were mock infected or infected with the P/V-CPI- mutant at an MOI of 10, and cultured in the presence of the indicated concentration of drugs. After 24 h, cell viability was determined by MTT assay as described in Methods section. (**B**) A549 cells were pretreated with 5 µM scriptaid or DMSO as a vehicle control for 12 h. Cells were mock infected or infected with the P/V-CPI- mutant at an MOI of 10, and cultured in the presence of 5 µM scriptaid or DMSO. Cell viability was determined by annexin V (left panel) and PI staining (right panel) at 24 hpi. (**C**) H1299 (left panel) or A549 (right panel) cells were pretreated with 20 µM scriptaid or DMSO for 12 h before mock infection or P/V-CPI- infection at an MOI of 10. After culturing in the presence of 1 µM scriptaid or DMSO for 24 h, cytotoxicity was determined by CytoTox-Glo assay. Values in all panels are the mean of three replicates normalized to DMSO pretreated mock infected samples with error bars indicating standard deviation. ** and *** indicates *p*-value of <0.01 and <0.001 comparing DMSO versus HDAC inhibitor pretreated infected samples.

To determine the effects on cell viability of combining HDAC inhibitors with P/V-CPI- mutant infection, human non-small cell lung cancer H1299 cells were pretreated for 12 h with DMSO as a vehicle control, or with various concentrations of either SAHA or scriptaid. SAHA was the first HDAC inhibitor approved by the FDA and scriptaid is a structurally related novel pan-HDAC inhibitor [30]. Cells were then mock infected or infected with the P/V-CPI- mutant at a multiplicity of infection (MOI) of 10 plaque forming units (PFU)/cell for 24 h Cell viability was determined using MTT assays. As shown in Figure 1A, pretreatment with either HDAC inhibitor enhanced death induced by the P/V-CPI- mutant. For example, mock infected cells that were pretreated with 100 μM SAHA retained roughly 70% viability (left panel, Figure 1A), whereas cells pretreated with 100 μM SAHA and then infected with P/V-CPI- virus showed only ~35% viability. A similar enhancement of cell killing was observed with scriptaid pretreatment (Figure 1A, right panel).

We extended this study to another human non-small cell lung cancer cell line. A549 cells were pretreated with either DMSO as a control or with 5 μM scriptaid, followed by mock infection or P/V-CPI- infection. At 24 h post infection (hpi), cell viability was determined by staining with annexin V and propidium iodide (PI) followed by flow cytometry. As shown in Figure 1B, infected control cells showed ~35% and ~30% of the cell population being positive for annexin V (left panel) and PI staining (right panel), respectively. By contrast, scriptaid pretreated cells that were infected with P/V-CPI- virus showed ~60% and ~55% annexin V and PI positive staining.

To directly measure cytotoxicity, H1299 (Figure 1C, left panel) or A549 (Figure 1C, right panel) cells were pretreated with either vehicle control or scriptaid for 12 h, followed by mock infection or infection with P/V-CPI- virus. At 24 hpi, cytotoxicity was determined using a CytoTox-Glo assay which measures overall cell death. As shown in Figure 1C, P/V-CPI- infection of H1299 cells resulted in a ~9 fold increase in cell killing compared to control mock infected samples. By contrast, scriptaid pretreatment followed by P/V-CPI- infection resulted in a ~20-fold change in cytotoxicity. Similarly, in A549 cells (Figure 1C, right panel) control P/V-CPI- mutant infection resulted in a ~10-fold increase in cytotoxicity compared to mock infected samples, and this was enhanced to ~15-fold increase by scriptaid pretreatment.

**Figure 2:**
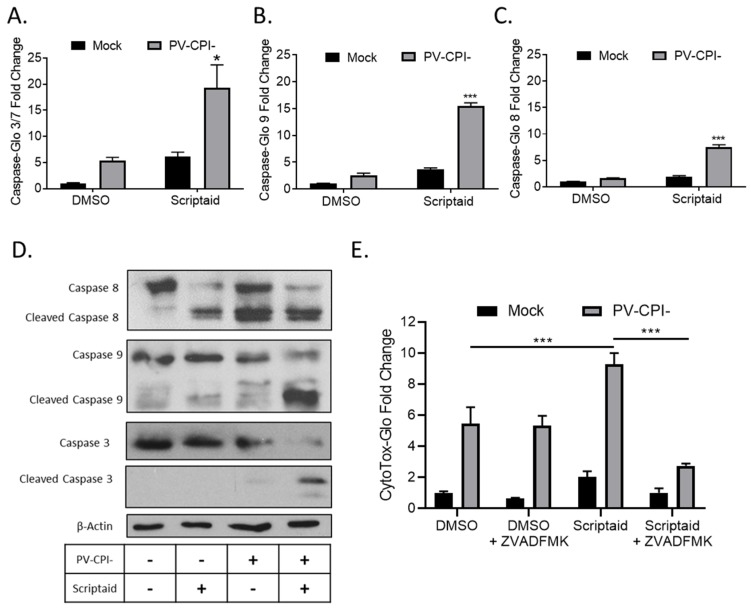
HDAC inhibitor enhances caspase activation in P/V-CPI- infected cells. (**A**–**C**) H1299 cells were pretreated with 20 µM Scriptaid or DMSO as a control for 12 h. Cells were then mock infected or infected with P/V-CPI- at an MOI of 10, and cultured for 24 h in media containing either 1 µM Scriptaid or DMSO. At 24 hpi, caspase activity was determined by Caspase-Glo-3/7 (**A**), -9 (**B**) or -8 (**C**) assays. Values are the mean of three replicates normalized to DMSO pretreated mock infected samples with * and *** indicating *p*-values of <0.05 and <0.001, respectively, comparing DMSO versus HDAC inhibitor pretreated infected samples. (**D**) A549 cells were treated as described for panels A-C. At 24 hpi, cells were harvested, and lysates were analyzed by western blotting for β-actin or for caspase-8, -9, and -3 cleavage products. (**E**) H1299 cells were pretreated with 20 µM scriptaid or DMSO for 12 h, and then mock infected or infected with P/V-CPI- at an MOI of 10. Cells were then cultured in media containing either 1 µM scriptaid or DMSO, with and without 50 µM of the pan-caspase inhibitor Z-VAD-FMK. At 24 hpi, cytotoxicity was determined by CytoTox-Glo assay. Values are the mean of three replicates normalized to DMSO pretreated mock infected samples with error bars indicating standard deviation and *** indicating *p*-values of <0.001.

To measure cellular caspase activity, H1299 cells were pretreated with 20 μM Scriptaid for 12 h followed by mock infection or infection at high MOI with P/V-CPI-. At 24 hpi, caspase activity was determined using caspase-glo assays. As shown in Figure 2A, effector caspases 3/7 were increased ~5-fold by Scriptaid treatment of mock infected cells, but were increased ~20-fold compared to control samples when Scriptaid pretreated was combined with P/V-CPI- infection. In these same samples, activities of the extrinsic pathway initiator caspase 8 and intrinsic pathway initiator caspase 9 were increased ~9-fold and ~15-fold, respectively, in cells treated with scriptaid and infected with P/V-CPI- virus compared to mock infected control samples (Figure 2B,C).

To confirm the above results, western blots were used to measure caspase cleavage products in lysates from A549 cells that were pretreated with scriptaid, followed by P/V-CPI- infection. As shown in Figure 2D, scriptaid pretreatment followed by virus infection induced higher levels of cleaved caspases-9 and -3 compared to treatment with inhibitor alone or P/V-CPI- alone. Increases in caspase-8 cleavage products following combined treatment was less clear, consistent with the lower induction of enzyme levels shown in Figure 2B. The increase in caspase activity after scriptaid pretreatment was responsible at least in part for the increases in cell death. This is evident in Figure 2E, where culturing cells with a pan-caspase inhibitor Z-VAD-FMK greatly reduced the level of killing in scriptaid-pretreated cells infected with P/V-CPI- virus.

Taken together, these data from two airway cancer cell lines and three different cytotoxicity assays indicate that scriptaid pretreatment followed by P/V-CPI- infection leads to increases in caspase-9 and -3/7 activity and to increases in cell killing through caspase-dependent pathways.

### 3.2. Double Stranded RNA Contributes to scriptaid-Mediated Enhancement of Cell Killing by the P/V-CPI- Virus

**Figure 3:**
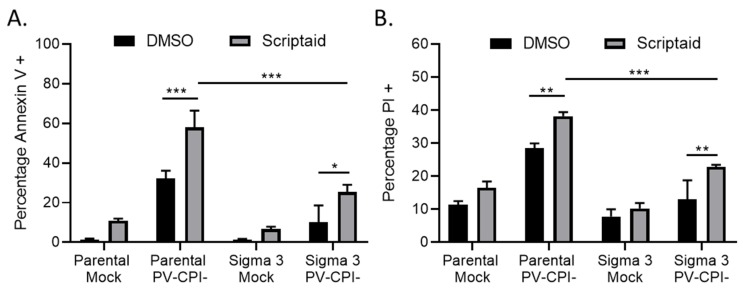
dsRNA contributes to enhanced death in cells treated with HDAC inhibitor and P/V-CPI- infection. (**A**, **B**) Parental A549 cells and A549 cells expressing reovirus sigma 3 protein were pretreated with 20 µM Scriptaid or DMSO for 12 h. Cells were mock infected or infected with P/V-CPI- virus at an MOI of 10, and cultured in the presence of 1 µM Scriptaid or DMSO control. Cell viability was determined by Annexin V (**A**) and PI (**B**) staining at 24 hpi. Values are the mean of three replicates with error bars indicating standard deviation. ** and *** indicating *p*-values of <0.01 and <0.001, respectively.

We have previously shown that strong induction of proinflammatory cytokines by the P/V-CPI- virus is inhibited in a A549 cell line that was engineered to constitutively express the reovirus sigma3 protein—a viral protein known to specifically bind to and sequester double stranded RNA (dsRNA) [31,37]. Given that viral dsRNA can be an inducer of apoptosis [38], we tested the hypothesis that if dsRNA was involved in scriptaid-mediated enhancement of P/V-CPI- killing, cell death would be reduced in sigma 3-expressing cells. Parental A549 cells and A549-sigma 3 cells were pretreated with scriptaid for 12 h before mock infection or high MOI infection with P/V-CPI- virus. Cells were analyzed for Annexin V and PI staining at 24 hpi. As shown in Figure 3, both Annexin V (panel A) and PI (panel B) staining was enhanced in P/V-CPI-infected parental A549 cells by prior treatment with scriptaid. Most importantly, staining for both of these cytotoxicity markers was significantly reduced in A549 cells expressing sigma 3 protein, indicating dsRNA plays a role in the scriptaid-mediated enhancement of P/V-CPI- cell death.

### 3.3. Scriptaid Pretreatment Enhances Killing of Lung Cancer Cells Infected with LACV and VSV

**Figure 4:**
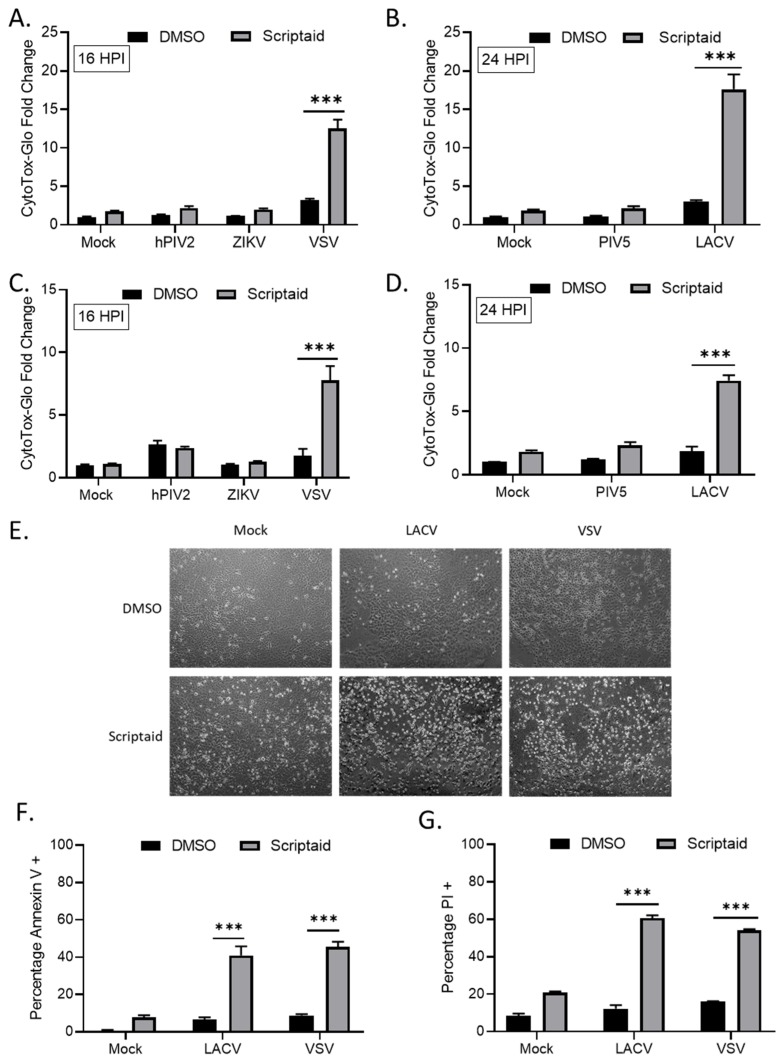
HDAC inhibitor pretreatment enhances cell death by other RNA viruses. (**A**–**D**) H1299 (**A**,**B**) or A549 (**C**,**D**) cells were pretreated with 20 µM scriptaid or DMSO for 12 h. Cells were then either mock infected or infected with the indicated viruses at an MOI of 10. After culturing in the presence of 1 µM scriptaid or DMSO for either 16 (**A**,**C**) or 24 (**B**,**D**) h, cytotoxicity was determined by CytoTox-Glo assay. hPIV2, human parainfluenza virus 2; ZIKV, Zika virus; VSV, vesicular stomatitis virus. LACV, La Crosse virus. Values are the mean of three replicates normalized to DMSO pretreated mock infected samples, with error bars indicating standard deviation. ** and *** indicating *p*-values of <0.01 and <0.001, respectively. (**E**–**G**) A549 cells were pretreated with 20 µM scriptaid or DMSO and either mock infected or infected with LACV or VSV at MOIs of 10. After culturing in the presence of 1 µM scriptaid or DMSO for 16 h, cells were imaged at 10× magnification, and representative bright field images are shown (**E**). Cell viability was determined by annexin V (**F**) and PI (**G**) staining.

We tested the hypothesis that HDAC-mediated enhancement of *p*/V-CPI- cell killing would extend to other cytoplasmic-replicating RNA viruses. A549 or H1299 cells were pretreated with Scriptaid for 12 h prior to mock infection or high MOI infection with hPIV2, ZIKV, VSV, WT PIV5 or LACV. Due to the large virus-induced cytopathic effects seen at late times pi, the cells infected with hPIV2, ZIKV and VSV were analyzed at 16 hpi by CytoTox-Glo assay. For WT PIV5 and LACV, cells were analyzed at 24 hpi. As shown in Figure 4, Scriptaid pretreatment enhanced killing of both H1299 and A549 cells by VSV (panels A and C) and LACV (panels B and D) infections, but this was not seen after infection with hPIV2, ZIKV or WT PIV5. Microscopic analysis of VSV and LACV infected cells showed relatively little cytopathic effect (e.g., cell rounding) in control treated cultures, but this was greatly enhanced in scriptaid-pretreated cells (Figure 4E). The scriptaid-mediated enhancement of VSV and LACV cell killing was confirmed by analysis of Annexin V and PI staining (Figure 4F,G). 

### 3.4. HDAC Inhibitor Pretreatment Downregulates IFN-β Production and Enhances Spread of the P/V-CPI- Mutant

**Figure 5:**
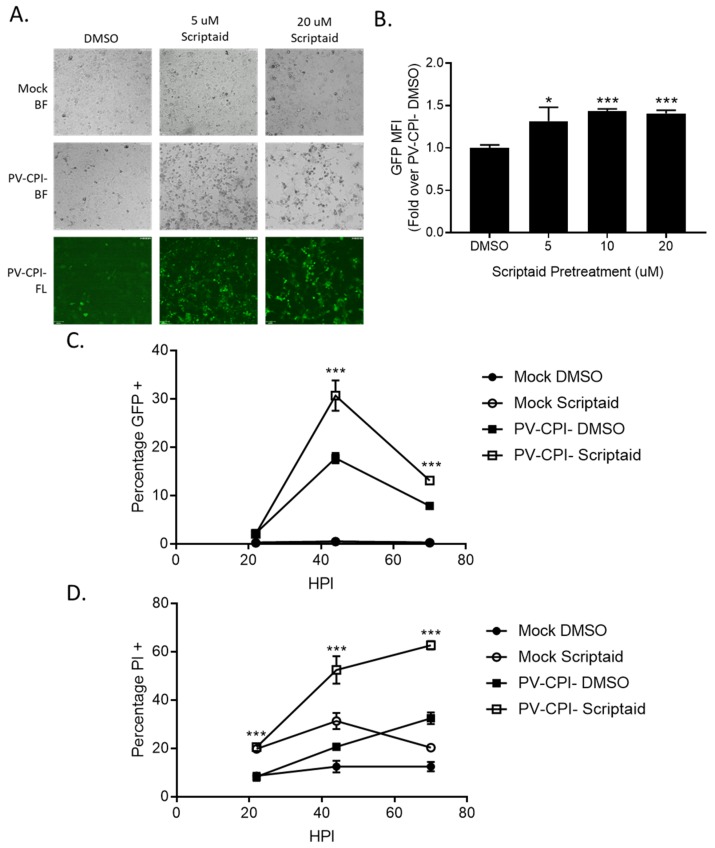
Pretreatment of cells with an HDAC inhibitor increases P/V-CPI- GFP expression and promotes low MOI virus spread. **(A**,**B**) H1299 cells were pretreated with the indicated concentrations of scriptaid or control DMSO for 12 h. Cells were then mock infected or infected with P/V-CPI- at an MOI of 10. After 24 h of culture in media containing either 1 µM scriptaid or DMSO, 20× bright field (BF) or fluorescence (FL) images of cells were captured (**A**). Alternatively, cells were analyzed by flow cytometry for GFP expression (**B**). Values are expressed as fold change over DMSO treated virus infected cells set at 1.0. (**C,D**). A549 cells were pretreated with 20 µM scriptaid or DMSO for 12 h. Cells were then mock infected or infected with the P/V-CPI- mutant at an MOI of 0.05, and cultured in media containing 1 µM scriptaid or DMSO. At the indicated hpi, cells were harvested and analyzed by flow cytometry for GFP expression (**C**) or PI staining (**D**). For all panels, error bars indicate standard deviation. * and *** indicates *p*-values of <0.05 and <0.001, respectively.

During the course of our studies, we observed that infected cells pretreated with scriptaid had higher GFP expression derived from the P/V-CPI- genome compared to infected control untreated cells. This is evident in Figure 5A, where microscopy analysis of infected H1299 cells showed brighter green fluorescence in cultures of scriptaid pretreated P/V-CPI- infected cells compared to the DMSO pretreated P/V-CPI- infected cells. When analyzed by flow cytometry, 10 μM scriptaid pretreatment reproducibly resulted in ~1.5-fold increase in GFP intensity during high MOI P/V-CPI- infections compared to vehicle treated cells (Figure 5B).

To determine if scriptaid pretreatment alters P/V-CPI- spread through a population of cells, A549 cells were infected at an MOI of 0.05 and cells were analyzed over time for GFP expression (Figure 5C) and PI staining (Figure 5D). At 45 hpi, control cultures of P/V-CPI- infected cells showed GFP expression and PI staining in ~15% and 20% of cells, respectively. By contrast, scriptaid pretreatment resulted in GFP expression and PI staining in 30% and 50% of the cells. At later times after infection, GFP expression in the cell population decreased in both control and scriptaid treated samples, likely due to increased cell death. These data indicate that scriptaid pretreatment relieves a restriction on low MOI spread of P/V-CPI- through a cell population. 

**Figure 6:**
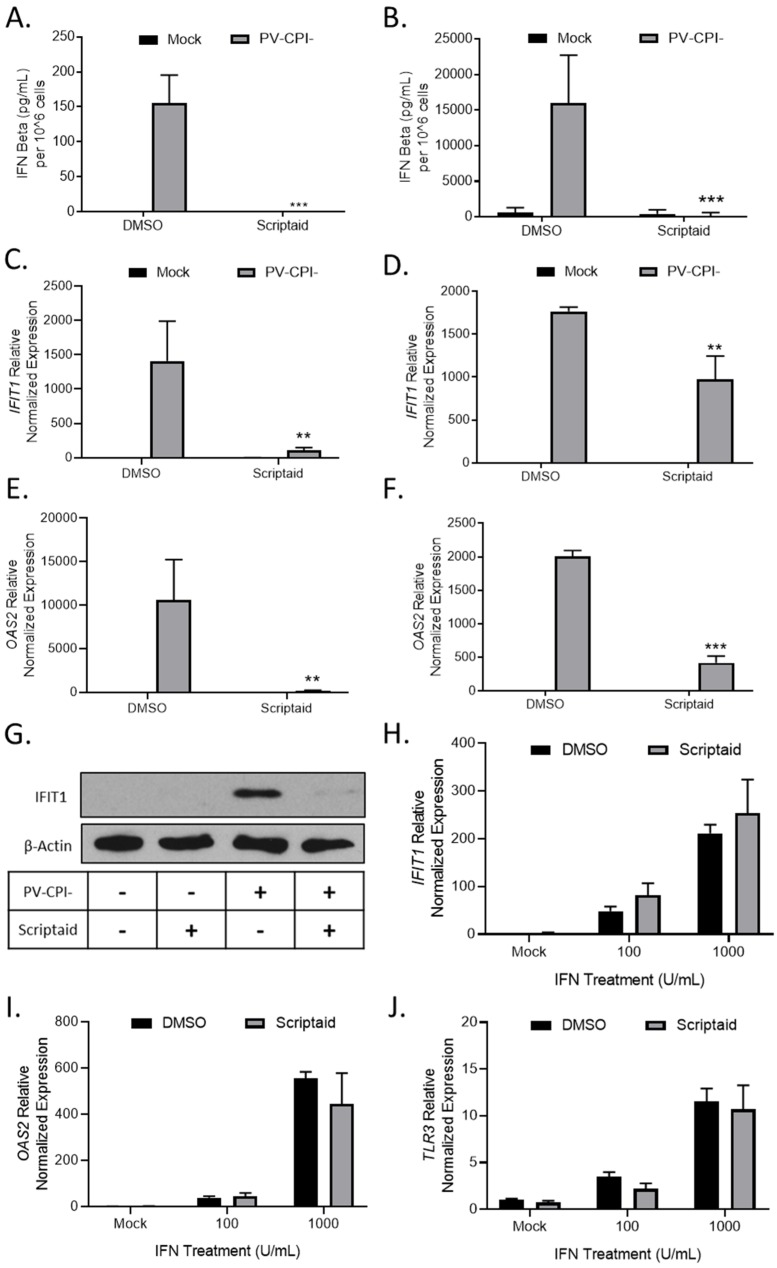
HDAC inhibitor pretreatment decreases P/V-CPI- induction of IFN-β and ISG expression. (**A**–**G**) H1299 (**A,C,E**) or A549 (**B,D,E**) cells were pretreated with 20 µM scriptaid or DMSO for 12 h. Cells were then mock infected or infected with P/V-CPI- at an MOI of 10 and cultured in media containing either 1 µM scriptaid or DMSO. At 24 hpi, media was collected and analyzed for IFN-β amounts by ELISA (**A,B**). Total cellular RNA was extracted and evaluated for *IFIT1* (**C,D**) and *OAS2* (**E,F**) expression levels by RT-qPCR. (**G**) H1299 cell lysates were analyzed for levels of IFIT protein by western blotting. (**H**–**J**) H1299 cells were pretreated with 20 µM scriptaid or DMSO for 12 h. Cells were then either mock treated or treated with 100 or 1000 U/mL of universal type 1 IFN. At 24 hpi, total cellular RNA was extracted and evaluated for *IFIT1* (**H**), *OAS2* (**I**), and *TLR3* (**J**) expression levels by RT-qPCR. For all panels, error bars indicate standard deviation. ** and *** indicates *p*-values of <0.01 and <0.001, respectively.

Given our previous findings that P/V-CPI- is a potent inducer of IFN-β and is defective in blocking IFN signaling [16], we tested the hypothesis that scriptaid pretreatment enhanced virus spread by altering IFN responses. H1299 and A549 cells were pretreated with DMSO as a control or scriptaid, and then mock infected or infected with P/V-CPI- at an MOI of 10. At 24 hpi, media was collected and IFN-β levels were determined by ELISA. As shown in Figure 6A and B, mock infected cells produced minimal IFN-β levels, which were largely unaltered by scriptaid. As shown previously [16], P/V-CPI- infected control cells produced high amounts of IFN-β (Figure 6A,B). Most importantly, virus-induced IFN-β secretion was effectively eliminated from cells that had been pretreated with scriptaid.

To determine if scriptaid pretreatment altered IFN stimulated gene (ISG) expression in P/V-CPI-infected cells, H1299 and A549 cells were pretreated with DMSO or scriptaid and then infected at an MOI of 10 with P/V-CPI-. Expression of two ISGs was analyzed by qPCR at 24 hpi. As shown in Figure 6C and D, *IFIT1* and *OAS2* genes were induced by P/V-CPI- infection of control cells. Scriptaid pretreatment significantly reduced the expression of these ISGs after P/V-CPI- infection. Western blotting confirmed scriptaid pretreatment reduced IFIT1 protein levels in H1299 cells (Figure 6G).

The above described scriptaid-mediated reduction in ISG expression could be due to a direct altering of IFN signaling, or alternatively, be primarily due to the loss of IFN-β production which indirectly reduces ISG expression due to loss of autocrine/paracrine signaling. In the absence of virus infection, control and scriptaid-pretreated H1299 cells were induced with increasing levels of IFN and ISG expression was assayed by qPCR. As shown in Figure 6H–J, scriptaid pretreatment did not significantly alter the induction of *IFIT1*, *OAS2* or *TLR3* genes by exogenously-added IFN.

Taken together, these data support the conclusion that scriptaid pretreatment directly reduced IFN-β production, which in turn indirectly reduced ISGs expression, contributing to enhanced P/V-CPI- spread and cell death.

### 3.5. Scriptaid Treatment Reduces P/V-CPI-Induced Nuclear Localization of IRF-3

**Figure 7:**
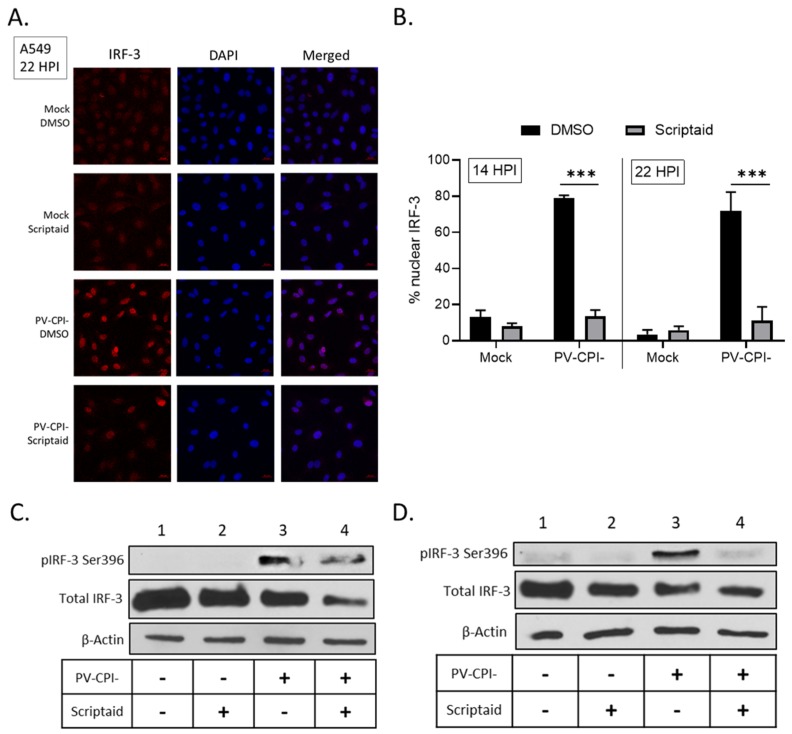
Effect of scriptaid treatment on P/V-CPI-induced IRF-3 nuclear localization and phosphorylation. (**A,B**) A549 cells were pretreated with 20 µM scriptaid or DMSO for 12 h. Cells were then mock infected or infected with P/V-CPI- at an MOI of 10 and cultured in media containing either 1 µM scriptaid or DMSO. IRF-3 immunostaining and DAPI nuclear staining was performed at 22 hpi and imaged at 40× magnification (**A**). Samples from the experiment displayed in panel A were used to determine the number of cells displaying intense nuclear staining as a percentage of the population (**B**). For each sample, five random fields were counted and averaged, with error bars denoting standard deviations. (**C,D**) A549 (**C**) and H1299 (**D**) cells were treated as described in panel A. At 24 hpi, cell lysates were evaluated for IRF-3 phosphorylated at Ser396, total IRF-3 and β-actin by Western blotting.

Following virus infection, IFN-β synthesis requires the phosphorylation and translocation of IRF-3 to the nucleus to initiate transcription of the *IFN-β* gene [39]. To determine if scriptaid treatment altered IRF-3 nuclear translocation, A549 cells were treated with DMSO or scriptaid and then infected at high multiplicity with P/V-CPI-. At 22 hpi, IRF-3 location was examined by immunofluorescence. As seen in the representative images in Figure 7A, mock infected cells showed diffuse cytoplasmic IRF-3 staining which was largely unaltered by scriptaid treatment. Consistent with previous results [31,34] and the strong induction of IFN-β synthesis by P/V-CPI-, nearly all P/V-CPI-infected cells showed intense nuclear IRF-3 staining. Most importantly, in the case of most cells pretreated with scriptaid, P/V-CPI- infection did not produce intense IRF-3 nuclear staining, but rather the staining was seen in a pattern resembling mock infected samples. Quantification of multiple microscopy images showed that ~70–80% of P/V-CPI- infected cells showed nuclear IRF-3 staining at either 14 or 22 hpi, which was reduced to ~10% by scriptaid pretreatment.

IRF-3 is phosphorylated in the cytoplasm prior to nuclear translocation to activate the *IFN-β* gene [39]. Western blotting was carried out to determine if scriptaid treatment altered P/V-CPI-induced phosphorylation of IRF-3 at residue Ser396. As shown in Figure 7C and D, P/V-CPI- infection induced phosphorylation of IRF-3 (lane 3), consistent with strong induction of IFN-β synthesis. In lysates from scriptaid-treated P/V-CPI- infected cells, there was a reduction in IRF-3 phosphorylation but this was typically not completely removed.

### 3.6. Post-Infection Treatment of P/V-CPI-Infected Cells with a Panel of HDAC Inhibitors Reveals Two Cell Killing Profiles

**Figure 8:**
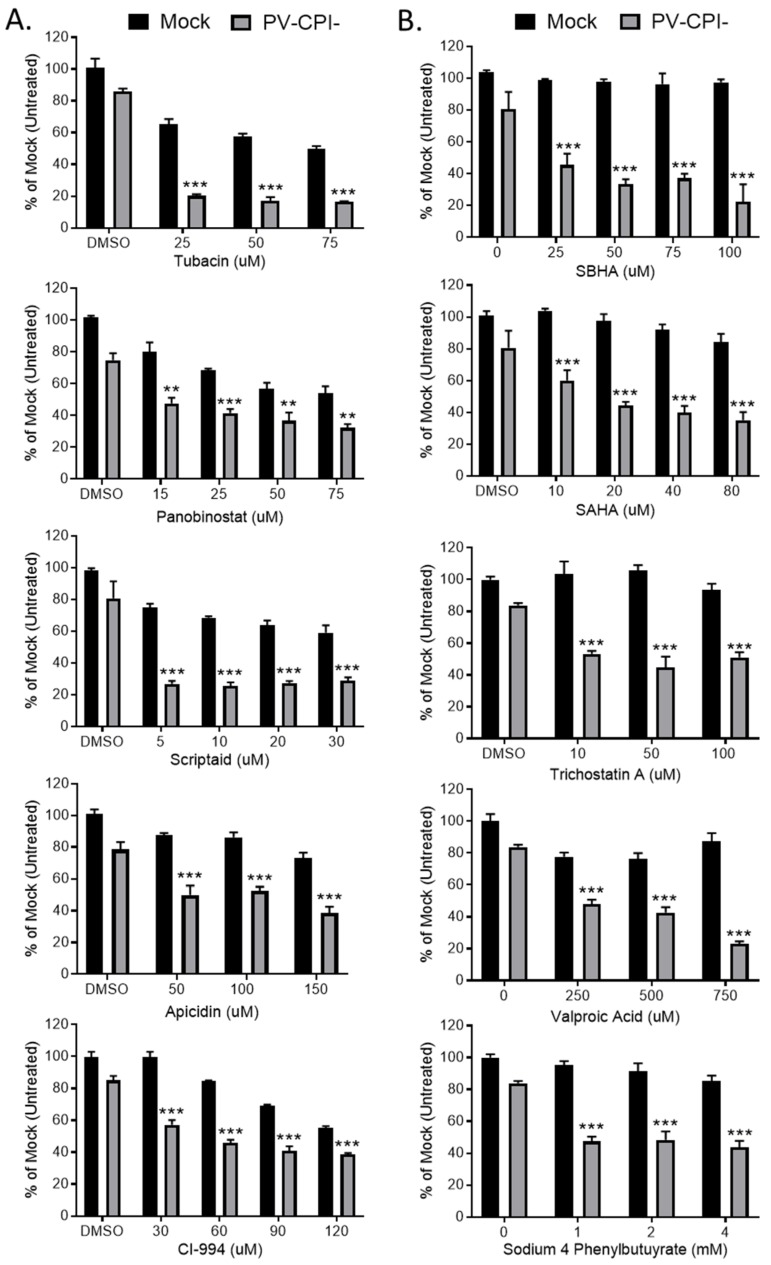
Differential effect of various HDAC inhibitors on killing of mock infected and P/V-CPI- infected human laryngeal cancer cells. (**A**,**B**) HEp-2 cells were mock infected or infected with P/V-CPI- at an MOI of 10 PFU/cell. At 12 hpi, cells were treated with indicated concentrations of HDAC inhibitors or solvent control. At 24 h post treatment, cell viability was determined by MTT assay. Graphs were organized based if HDAC inhibitors reduced viability of mock infected cells (panel A) or if the inhibitors did not reduce viability of mock infected cells (panel B). Values are the mean of three replicates with ** and *** indicating *p*-values of <0.01 and <0.001, respectively, comparing DMSO versus HDAC inhibitor treated infected samples. Error bars indicate standard deviation.

We extended our analysis of the effect of 10 HDAC inhibitors on P/V-CPI- killing of HEp-2 cells, a human laryngeal cancer cell line. In addition, we tested the hypothesis that treating cells with these inhibitors after P/V-CPI- infection would enhance cell death. Cells were mock infected or infected at high MOI with P/V-CPI-, and at 12 hpi were treated with a range of concentrations of 10 different HDAC inhibitors shown in Figure 8. At 24 h post treatment, cell viability was determined by MTT cell viability assay. The results shown in Figure 8 are organized into two groups which reflect the differential effect of HDAC inhibitors on the viability of mock infected versus P/V-CPI-infected cells. Figure 8 panel A shows examples of 5 HDAC inhibitors which enhance P/V-CPI- killing but also greatly reduce the viability of mock infected control cells. For example, treatment of cells with 50 μM Tubacin or Panobinostat resulted in ~10% and 40% viability when coupled with P/V-CPI infection, respectively. However, mock infected cell viability was also reduced to ~50–60% by 50 μM of the HDAC inhibitors (Figure 8A). By contrast, Figure 8 panel B shows examples of drugs such as SBHA, SAHA, and Trichostatin A which had a very modest effect on the viability of mock infected control cells even at high concentrations of the drug (e.g., 100 μM), but showed strong loss of viability in P/V-CPI- infected cells. These data suggest that not all HDAC inhibitors will function optimally with P/V-CPI- infections, and the relative effectiveness of each drug may differ between different human airway cancer cells.

## 4. Discussion

We have previously shown that infection with the cytoplasmic-replicating RNA virus P/V-CPI-can sensitize airway cancer cells to chemotherapeutic DNA damaging agents through the modulation of DNA damage response pathways [26]. This prior finding led us to test the hypothesis that P/V-CPI- infection would also sensitize cancer cells to treatment with other chemotherapeutic agents that alter gene expression, DNA metabolism and stress responses. Here we show that airway cancer cells treated with a variety of HDAC inhibitors show dose-dependent enhanced cell killing by the P/V-CPI- mutant virus as well as two other negative strand RNA viruses. This enhanced killing is due to the upregulation of caspase-dependent death pathways that include, at least in part, the response to dsRNA. In addition, treatment with the HDAC inhibitor scriptaid repressed IFN-β production which is normally potently induced by P/V-CPI- infection and led to increased viral spread through the cell population.

HDAC inhibitors have shown promise as chemotherapy agents. While four of these chemical inhibitors have been approved by the FDA for hematologic cancers, there are disadvantages on their use as single agents such as less success in solid tumor therapies [40]. Therefore, numerous ongoing clinical trials are investigating HDAC inhibitors and additional forms of cancer therapies, such as other chemotherapies, radiation, and immunotherapies [30]. One such promising combination therapy is HDAC inhibitors and oncolytic viruses. Due to the pleiotropic effects of HDAC inhibitors on various pathways, their mechanism of action is often complex when coupled with oncolytic viruses. For example, valproic acid was shown to act synergistically with the DNA viruses adenovirus, herpes virus, and vaccinia virus; however, it antagonized the potency of a different strain of adenovirus [41,42,43,44,45]. The proposed mechanisms of enhanced cell death with HDAC inhibitors and DNA oncolytic viruses are very diverse, such as amplified viral replication (including increased viral entry due to increased receptor expression), reduced antiviral responses, increased apoptosis or autophagy, increased NF-kB activity, increased cell cycle arrest, and increased oxidative stress [46,47,48].

Our study was prompted by an interest in how HDAC inhibitors could be used with a novel oncolytic virus based on a cytoplasmic replicating parainfluenza mutant. Shulak et al. [49] previously showed in prostate cancer cell lines that the combination of HDAC inhibitors with VSV infection resulted in an increase in virus replication and caspase dependent death, as well as a decrease in IFN-α and ISG expression. HDAC inhibitors were shown to induce NF-κB-regulated genes and increased NF-κB dependent autophagy, which led to enhanced death. In pancreatic cancer cell lines, Ellerhoff and co-workers [50] found synergistic effects of HDAC inhibitors and measles virus infection on cell death, however there was no alterations in virus growth or in IFN signaling pathways. Our results with P/V-CPI- and airway cancer cells differs from these two prior reports by showing that scriptaid pre-treatment: (1) enhanced killing through caspase-dependent pathways (versus autophagy for VSV; [49]), (2) promoted virus spread through a cell population, and (3) reduced IFN-β production and ISGs expression through a block at or upstream of IRF-3 nuclear translocation.

Our studies show the effect that an HDAC inhibitor can have on RNA virus activation of IFN pathways. P/V-CPI- is a potent inducer of IFN-β, due to alterations in both the V protein and the P protein component of the viral polymerase [16,34], and it is restricted in spread due to the paracrine and autocrine effects of IFN-β [25]. As reviewed by Eckschlager et al. [29] and Suraweera et al. [30], HDAC inhibitors have a tumor cell specificity and show minimal effects on healthy cells. Therefore, healthy cells should be able to produce IFN and induce an anti-viral state upon P/V-CPI- infection, thereby maintaining tumor specificity of our proposed combination therapy. Here we show that Scriptaid treatment reduced IFN-β production from P/V-CPI-infected cells to background levels, and enhanced low MOI growth and killing of a cell population. While prior work has shown that HDAC inhibition can in some cases alter IFN signaling [51], we showed that with this virus/cell system, HDAC inhibitors directly limited IFN induction but did not directly affect ISG expression. Virus induced IRF-3 nuclear translocation was blocked by scriptaid treatment, suggesting an alteration in cytoplasmic sensing or signaling versus more distal effects on nuclear transcription of the *IFN-β* gene. While IRF-3 phosphorylation at a key regulatory site was reproducibly decreased by scriptaid treatment, it is not clear that this was sufficient to account for the nearly complete loss of IFN-β production. Future work will address the specific step in IFN-β induction altered by HDAC inhibition. 

HDAC inhibitors can regulate expression of proteins involved in apoptosis, including an increase in pro-apoptotic protein family members, e.g., Bid, Bim, Bmf, Bad, and Noxa [52,53,54,55]. Conversely, HDAC inhibitors can result in a decrease in anti-apoptosis proteins such as Bcl-2 and Bcl-w, as well as in select caspase inhibitors, such as X-linked inhibitor of apoptosis (XIAP), survivin, and cellular FLICE-like inhibitory protein (c-FLIP) [56,57,58]. Signals that activate the extrinsic apoptosis pathway have also been shown to increase following HDAC inhibitor treatment [59,60,61]. For example, Nakata et al. [62] found levels of death receptor 5 to be upregulated following HDAC inhibitor treatment, leading to increased caspases -8, -10, -9, and -3 activation and apoptosis induced by Tumor Necrosis Factor (TNF) related apoptosis ligand (TRAIL). While at this point it is unclear how HDAC inhibitors promote P/V-CPI-medicated increases in caspases, there is a potential mechanism linked to scriptaid-induced decreases in cellular inhibitors of apoptosis. This is supported by our previous findings that expression of cIAP-1, XIAP, and survivin are all decreased by P/V-CPI- infection and that chemical inhibition of survivin (with YM155) or XIAP (with Embelin) enhanced killing of a P/V-CPI- persistently infected cell line [26].

dsRNA produced during the course of virus infection can be a potent inducer of cell death [38], through pathways that can involve both caspase-8 and -9 [63]. Here we show that P/V-CPI-induced killing is decreased in an A549 cell line engineered to express the reovirus sigma 3 protein which sequesters dsRNA, and this decreased killing is seen in both untreated and scriptaid treated A549-sigma 3 cells. This suggests that scriptaid does not activate alternative death pathways, but rather amplifies death pathways already activated by P/V-CPI-.

Scriptaid treatment promoted airway cancer cell killing by P/V-CPI-, but also by two other negative strand RNA viruses we tested – LACV and VSV. While the mechanism by which WT PIV5 can replicate to very high levels in most cell types but not induce a cytopathic effect is currently unknown, it was nonetheless expected that HDAC inhibitors would not alter this phenotype in airway cancer cell lines. Scriptaid treatment had a very strong effect on LACV cytopathic effect. What was surprising, however, was our finding that HDAC inhibitors did not accelerate killing by infection with hPIV2 or ZIKV, both of which are associated with strong cytopathic effects. Since a number of groups are proposing the use of ZIKV as an oncolytic vector [64], this result may warrant further in-depth study to investigate variables such as HDAC inhibitors other than scriptaid, other cancer cell types, or the length of treatment and infection.

The timing of HDAC inhibitor treatment can play a role in the effectiveness of the combination treatment. For example, pretreatment before viral infection can improve viral yields, however concurrent treatment eliminated this effect [41,65]. Due to the multifaceted nature of HDAC inhibitors, our studies showcase the necessity of personalized medicine by characterizing tumors to better indicate the proper treatment options. Which therapies to combine, the timing and length of treatments are imperative factors that need to be considered when utilizing a combination approach with HDAC inhibitors and oncolytic viruses.
